# Interventions for the management of snakebite envenoming: An overview of systematic reviews

**DOI:** 10.1371/journal.pntd.0008727

**Published:** 2020-10-13

**Authors:** Soumyadeep Bhaumik, Deepti Beri, Zohra S. Lassi, Jagnoor Jagnoor

**Affiliations:** 1 The George Institute for Global Health, University of New South Wales, Sydney, New South Wales, Australia; 2 The George Institute for Global Health, New Delhi, India; 3 Robinson Research Institute, The University of Adelaide, North Terrace, Adelaide, South Australia, Australia; Instituto Butantan, BRAZIL

## Abstract

**Introduction:**

Snakebite is a neglected tropical disease that leads to more than 120,000 deaths every year. In 2019, World Health Organization (WHO) launched a strategy to decrease its global burden by 2030. There is a range of issues around different interventions for the management of snakebite. Decisions around these interventions should be informed by evidence from systematic reviews (SR).

**Methods:**

An overview of SRs was conducted by searching 12 electronic databases, PROSPERO, contacting experts and screening the bibliography of included reviews. Screening, data extraction, and quality assessment (through AMSTAR-2) was done by at least two overview authors independently with discrepancies sorted by consensus. A narrative synthesis was conducted.

**Principle findings:**

The overview found 13 completed SRs that has looked at various aspects of management of snakebite envenomation. There was one SR on first aid, nine on effectiveness and safety of snake anti-venom (SAV), two on drugs to prevent adverse reactions due to SAV therapy, and one on surgical interventions for management of snakebite envenomation. All, except one, SR was appraised to have critically low confidence as per AMSTAR-2 Criteria. Evidence base was restricted to few studies for most interventions.

**Discussion:**

High quality evidence from SRs is required to inform guidelines and health system decisions which can bring down the burden of snakebite. The review indicates the need to fund high-quality SRs, evidence gaps and core outcome sets which can inform guideline recommendations, funding priorities for conduct of future trials. Variation in species distribution as well as intra-species variation in venom composition implies the need for conduct of region or, nation or state (sub-national) specific randomised controlled trials and SRs on different SAVs and their dosing regimens.

## Author summary

Snakebite is a neglected tropical disease which has received priority attention in the global health space with WHO setting a target to decrease death and disability due to snakebite to 50% by 2030. High quality systematic reviews can inform policy and practice. We searched 13 electronic databases and PROSPERO, screened reference lists, and contacted experts. We identified 13 completed systematic reviews which has reviewed effectiveness and safety for first-aid, snake anti-venoms, drugs to prevent adverse reactions and fasciotomy. Evidence for interventions often came from few studies. We judged confidence on the results of the systematic reviews using AMSTAR-2 and all except one review was judged to have critically low confidence. Evidence with respect to specific geographic settings and for many specific anti-venoms is unavailable at the synthesis level and at the primary study level. Evidence related to late adverse reactions, wound-related outcomes, quality of life, duration of hospitalisation, cost, and disability is scarcely reported. Funding evidence gap maps, systematic reviews and development of core-outcome sets based on the results of this overview and subsequent conduct of randomised controlled trials for snakebite envenomation is essential.

## Introduction

Snakebite is a neglected tropical disease which leads to more than 120,000 global deaths every year [1]. Disability, social and economic costs of snakebite is not well studied but overall burden of snakebite is understood to be grossly underestimated [2]. Snakebite is global in nature but it mostly affects rural and tribal communities in South Asia, Southeast Asia, and Africa [2]. A modelling study has estimated that inadequate provision of quality healthcare for snakebite affects 146.7 million people adversely [3].

Although 5.4 million snakebites occur annually, only about half of them leads to envenoming (the clinical condition after bite from a venomous snake). Snake venoms are highly complex and diverse, which show inter-species as well as intra-species variation [4–7]. Consequently, snakebite envenomation represents myriad clinical manifestations. These include, but not limited to, local wound, neurotoxic, renal, musculoskeletal, cardiovascular, haemostatic and mental health related manifestations [2, 4]. Management of snake envenomation involves first aid, management of local and systemic effects followed by management of complications and follow-up for addressing any sequalae or disability [4]. Snake anti-venom (SAV) is the only specific intervention that is required, but SAVs are of various types and there is substantial debate on not only its dosage and frequency but also, in its design and suitability in different geographic regions and for different species.

In 2019, the World Health Organization (WHO) has released a comprehensive strategy which aims to decrease the burden of death and disability due to snake envenomation by 50% before 2030 [8]. Ensuring safe and effective treatment is one of the four key pillars which WHO has identified. We have previously analysed existing WHO guidelines for management of snakebites and found poor methodological rigour in its development [9]. The WHO Guidelines were not based on systematic search, appraisal, and grading of evidence. Using evidence from high-quality systematic reviews (SRs) is crucial for decision-making. An overview of SRs will not only serve as a "single window front-end" on the current evidence but will also help identify gaps at the evidence synthesis level of interventions for management of snakebite envenomation. An overview of SRs is a relatively new approach for evidence synthesis with research methodology and guidance around it evolving [10]. It essentially involves systematically searching, appraising, and synthesising the results of related and relevant SRs on a single topic to support decision making by clinicians, policy makers, and guideline developers.

## Methods and analyses

The protocol for the overview was registered prospectively in PROSPERO (CRD42018073048). The PRISMA checklist is provided in **S1 Table**.

### Justification of overview of systematic reviews as the right approach for the study

We followed the Cochrane’s Comparing Multiple Interventions Methods Group Editorial Decision Tree to establish whether our review would better fit an overview design or an intervention SR design, with or without a network meta-analysis [11]. The overview of SRs is an appropriate study design for our research topic because we did not intend to compare multiple interventions to draw inferences about the comparative effectiveness of the interventions but intended to summarise the available evidence on different interventions for management of snakebite envenoming.

### Criteria for considering reviews for inclusion

We included studies which met the following criteria:

○ **Study Design:** SR, irrespective of the design of the individual studies included by them, irrespective of whether they have conducted a meta-analysis or not.
○ **Population:** SRs that have included studies with patients being treated for snakebite envenoming (irrespective of the snake species and irrespective of the age and sex of the participants or the setting).
○ **Interventions:** SRs that have included any kind of medical, surgical or complementary or alternative therapies that can be used as a single intervention or concurrently with others, irrespective of the comparator.
○ **Primary Outcomes**
  1. All-cause mortality.
  2. Any specific type of mortality (including but not limited to death due to neuromuscular paralyses or coagulopathy or cardiovascular shock, acute kidney injury).
  3. Early adverse reaction (immediate or anaphylactic reaction and/or early anaphylactoid reaction (archetypal use)- as defined by systematic review authors).
  4. Late adverse reactions to snake anti-venoms or serum sickness (as defined by the systematic review authors).
  5. Major Complications including but not limited to major haemorrhage, paralysis, muscle loss or kidney failure after snakebite (as defined by the systematic review authors).
  6. The proportion of wounds that have healed/are infection free or validated cosmetic outcome scores for wounds.
  7. Mental health-related outcomes (as defined by the systematic review authors).
○ **Secondary Outcomes**
  1. Duration of hospitalization
  2. Quality of life
  3. Any cost-related outcome
  4. Any other wound-related outcome (including but not limited to necrosis)
  5. Death or disability as composite outcome (as defined by systematic review authors)
○ If there was an update, we included only the latest version.
○ We included SRs irrespective of language or date of publication

### Search methods for identification of reviews

#### Electronic database

We searched Ovid MEDLINE(R), Global Health, EMBASE, Cochrane Database of Systematic Reviews, Database of Abstracts of Reviews of Effects, Cochrane Clinical Answers, Cochrane Central Register of Controlled Trials, Cochrane Methodology Register, Health Technology Assessment, NHS Economic Evaluation Database, APA PsycInfo, CINAHL by EBSCO-Host, and the Campbell Library. We also conducted supplementary search on Scielo (https://www.scielo.org/) for additional coverage of potential Spanish and Portuguese literature from Latin America. Detailed search strategy for all databases (updated 16^th^ May 2020) including the supplementary Search on Scielo (updated 04^th^ August 2020) is provided in **S1 Text**.

#### Search for grey literature

We contacted experts working in the domain of snakebite. We also searched PROSPERO, and the bibliographies of included SRs (found by other methods), to identify other SRs on the topic.

### Selection of reviews

In the first phase, two authors (SB and DB OR ZL) independently screened the studies retrieved based on titles and/or abstracts and marked each record as “exclude” or “needs full text for evaluation”. Full texts of all studies marked as “needs full text for evaluation” by either of the two authors were obtained and reviewed independently by two authors for consideration of inclusion based on criteria discussed above. Disagreements were resolved by consensus.

### Data extraction and management

Two authors (SB and DB or ZL) independently extracted data. We did not contact the authors of SRs, or authors of individual studies, for any clarification or missing data. Disagreements were resolved by consensus between two authors (SB and DB or ZL). We extracted data using a pre-designed data extraction sheet.

### Data synthesis

We narratively synthesised the results of the SRs. No additional quantitative analyses (additional indirect comparisons or network meta-analyses) or critical appraisal of studies included in SRs were conducted.

We provide a narrative description of the summary results from the included SRs. When there was an overlap between two SRs (i.e. had included same studies), we abstracted both the results, compared and contrasted them and reported both. If meta-analysis was conducted, the summary statistics is abstracted and reported, but in absence of meta-analysis, we present the summary results of the included studies. Unless otherwise mentioned all values correspond to 95% confidence interval (CI). We grouped studies for synthesis based on intervention types.

### Assessment of methodological quality of included SRs

Quality assessment for included SRs was done independently by two authors (SB and DB or ZL) using the AMSTAR– 2 [12] criteria and discrepancy, if any, was resolved by consensus. AMSTAR-2 is an internationally accepted tool for assessment of quality of SR. The AMSTAR-2 assessment pertains to the conduct of SR and is independent of the quality of included primary studies.

The assessment of quality of included primary studies, if reported in included SRs is presented.

### Difference between protocol and actual conduct of overview

As a matter of transparency, we note some protocol deviations during the conduct of the overview. Death or disability as composite outcome and any other wound-related outcome were not a priori outcomes noted in the protocol. These were added to capture additional evidence reported in SRs which could be useful for decision making. We searched 13 electronic databases, much more than originally planned. We had originally planned to search TOXLINE which is no longer a separate subset and relevant records subsumed within PubMed.

## Results

### Search results

We retrieved 76 records from search in electronic databases, 28 records in PROSPERO and two by citation screening in the original search. We removed duplicates (n = 30) and after screening following titles and abstracts (56 articles excluded) we retrieved 20 full texts from the original search strategy. For the supplementary search for Latin American literature, we retrieved 38 records with no duplicates and after screening, assessed four full texts.

Overall, we evaluated 24 full texts and finally included 13 completed SRs [13–25]. We identified three ongoing SRs which have protocol available in PROSPERO or are published [26–28] which meet our inclusion criteria.

**Fig 1:**
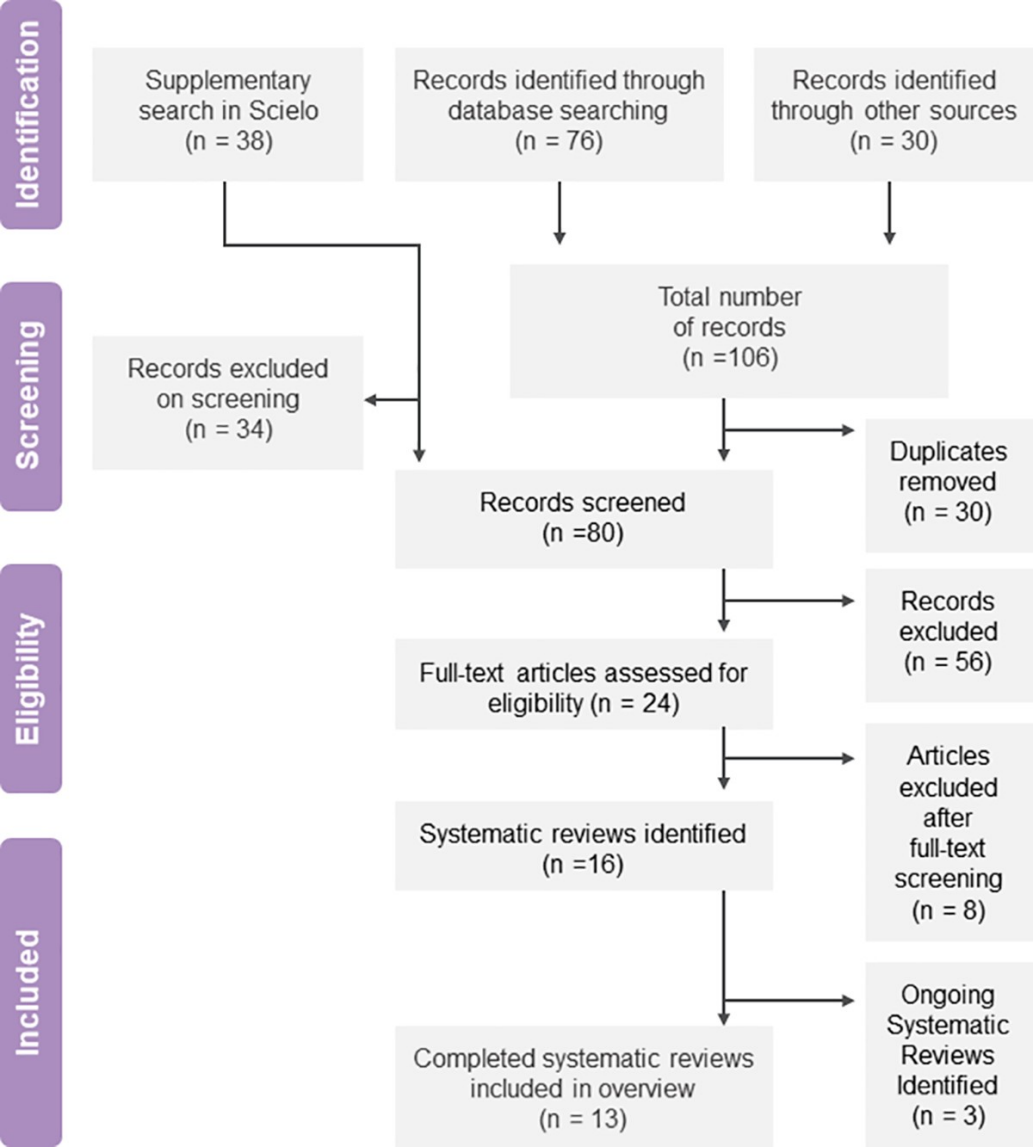
PRISMA flowchart for selection of SRs in the overview.

**Fig 1** shows the PRISMA flowchart documenting the process. Reasons for exclusion at full-text phase are mentioned in **S2 Table**.

### Description of included systematic reviews

The three ongoing SRs study effectiveness of SAV on neuromuscular paralysis [26], interventions for managing thrombotic microangiopathy due to snakebite [27], and the role of therapeutic plasma exchange in acute care (with a planned subgroup analysis for snakebite) [28].

We found 13 completed SRs. Characteristics of included SRs are summarised in Table 1.

**Table 1:** Characteristics of included systematic reviews.

NAME	Review Objective	Types of Study Design	Population & Setting	Intervention	Types of Comparator	Outcome	Date of Last Search
FIRST AID FOR SNAKEBITE							
Avau 2016	To summarize the best available evidence concerning effective and feasible first aid techniques for snakebite.	1. (quasi or non-) randomized controlled trials, controlled before and after studies or controlled interrupted time series. 2. Observational studies of the following types were also included: cohort and case-control study, controlled before and after study or controlled interrupted time series	Studies concerning people with snakebites or healthy volunteers with “mock” snakebites	Interventions for the first aid management of snakebites that can be applied by laypeople without medical background. Interventions for the management of snakebites that are not feasible to be performed in a first aid setting where laypeople are the first aid providers were excluded.	The interventions to any other first aid intervention or no intervention	(1) survival, functional recovery, pain, complications, time to resumption of usual activity, restoration of the pre-exposure condition, time to resolution of symptoms or other health outcome measures (including adverse effects) for studies involving snakebite victims, (2) spread of mock venom for studies investigating the efficacy of pressure immobilization and (3) quality of the bandage applied and tension generated for studies investigating the feasibility of pressure immobilization.	March 2016
EFFECTIVENESS AND SAFETY OF SNAKE ANTI-VENOM: SPECIES OR GENUS SPECIFIC SYSTEMATIC REVIEWS							
Johnson 2008	To analyse the literature concerning the utilisation of Crotalidae polyvalent immune fab (ovine; FabAV) in children following Crotalinae envenomation	Human case reports and studies	Paediatric patients less than 18 years of age **Setting:** not specified	Crotalidae polyvalent immune fab	not mentioned	not specified	Feb 2008
Lavonas 2009	To characterize the reported response to FabAV therapy of patients suffering severe crotaline envenomation.	All article types were considered, including prospective clinical trials, cohort and non-cohort case series, single case reports, review articles, editorials, commentaries, published abstracts, and letters-to-the-editor	**Victims of** North American severe crotalid envenomation 1. "severe" envenomation as defined in the US FDA-approved prescribing information for FabAV 2.Snakebite Severity Score (SSS)>7 3. Reviewer defined ""severity of envenomation based on the initial presentation,"	Treatment with FabAV	Not specified	1. "initial control" of a specific venom effect, (specific definition by SR author). 2. initial control of coagulopathy (specific definition by SR author). 3. Initial control of the envenomation syndrome (specific definition by SR author). 4. Persistent severe venom effects 5. Recurrence or delayed onset of severe venom effects 6. Permanent sequelae of envenomation	July 2008
Schaeffer 2012	To evaluate the incidence of immediate hypersensitivity reactions and serum sickness reported in studies of patients treated with FabAV therapy after North American crotaline envenomation.	All prospective and retrospective cohort studies	All patients receiving FabAV therapy for North American crotaline envenomations Setting Not specified	FabAV therapy	NA	Immediate hypersensitivity and serum sickness incidence associated with FabAV administration; rehospitalization or death of a patient as a result of serum sickness	December 2010
Lavonas 2014	To estimate the proportion of patients with crotaline snake envenomation who are treated with Crotalidae polyvalent immune Fab (ovine) antivenom and who develop medically significant late bleeding	Retrospective observational studies, prospective observational studies, and clinical trials	Crotaline snake envenomation in United States No restriction placed on study setting; therefore, all studies based in EDs, hospital inpatient units, outpatient centers, poison centers, and combinations were considered	FabAV	Not specified	All late bleeding events (specific definition by SR author). Deaths due to late bleeding event	May 2012
Habib 2013	To review and re-analyse all published preclinical and clinical studies on envenoming and antivenom therapy conducted in West Africa to determine the effectiveness of antivenom therapy of carpet viper (Echis ocellatus) envenoming	All observational, interventional and preclinical studies conducted in the region (or on antivenoms derived from the region)	Patients from Sub-Saharan/West African countries with carpet viper bites	Antivenom	Inappropriate or no antivenoms	Effectiveness of antivenoms in resolving features of carpet viper envenoming or curtailing mortality	March 2012
Lamb 2017	Identify all the anti-European Vipera spp antivenoms currently in clinical use and to seek data on comparative effectiveness and safety.	Publications (unspecified) pertaining to clinical outcome, including case reports	Europe Setting not specified	Anti-venom	not specified	Not specified	March 2016
EFFECTIVENESS AND SAFETY OF SNAKE ANTI-VENOM: BROAD NON-SPECIES OR NON-GENUS SPECIFIC SYSTEMATIC REVIEWS							
Das 2015	To evaluate the optimum dose (low vs. high) for snake antivenom (SAV)	RCTs	Patients having evidence of envenomation, irrespective of whether the bite was from a viper, cobra, or krait. Exclusion criteria were, presentation 24 h after the bite, history of any bleeding diathesis or any other previous neurological abnormality, and manifested allergy to the SAV. **Setting:** not mentioned	**Intervention: H**igh dose of SAV (not defined by review authors) **Co-intervention:** as an adjuvant to standard hospital treatment of snake bite. All methods of administration of SAV in all grades of envenomation (mild, moderate or severe) were considered.	low dose **SAV (not defined by review authors)**	**Primary outcome:** Mortality rate. **Secondary outcome:** -Time to normalization of CT; -Neurological complication rate; -Rate of other complications (acute renal failure [ARF], bleeding or disseminated intravascular coagulation [DIC], and shock); -Duration of hospital stay (days); -Adverse-events; -Cost-effectiveness.	August 2014
Maduwage 2015	To assess the effect of snake antivenom as a treatment for venom induced consumption coagulopathy in people with snake bite.	RCTs (with a placebo or no treatment arm)	People of any age with snake envenoming who have already developed snake venom induced consumption coagulopathy	Intravenous administration of snake antivenom regardless of the type of antivenom or the dose.	People not treated with antivenom	**Primary outcomes** •Mortality **Secondary outcomes** • Major haemorrhages •Time to improve clotting studies •Immediate systemic hypersensitivity reactions • Serum sickness	January 2015.
Potet 2019	To systematically collect and analyse the clinical data on all antivenom products now available in markets of sub-Saharan Africa.	All types of clinical data were eligible for inclusion: randomized controlled trials, case-control studies, observational cohort studies, case series, and programmatic data.	Sub-Saharan Africa. All patient populations of all ages were included. Studies reporting less than 10 patients per antivenom product were excluded.	Commercially available antivenom products	not specified	clinical data in terms of safety and effectiveness against the different species and envenoming syndromes.	February 2018
INTERVENTIONS TO MANAGE ADVERSE REACTIONS DUE TO SAV THERAPY							
Nuchprayoon 2000	To evaluate drugs given to prevent acute adverse reactions to horse serum antivenom, in relation to anaphylaxis and death.	Randomized or quasi-randomized controlled trials.	Patients treated for snake envenoming with horse serum antivenom, irrespective of the snake species.	1. Adrenaline versus no adrenaline. 2. Steroid versus no steroid. 3. Antihistamine versus no antihistamine.	As noted in intervention section	Primary - Death (from any cause). - Symptoms and signs indicating severe anaphylactic reaction (specific definition by SR author). Secondary - Early (anaphylactoid) reactions: urticaria, angioedema, bronchospasm. - Late (serum sickness type) reactions: fever, rash, arthritis, lymphadenopathy more than 5 days after antivenom.	Updated search on 29 March 2004 but newer studies were not included or excluded and original 1999 version of results retained
Habib 2011	To conduct a systematic review and meta-analysis of published data to assess the effect of pre-medication on the risk of EAR (early adverse reactions)	RCT or cohort study designs	Patients with early adverse reaction following antivenom administration in snakebite No regional restriction	antivenoms + pre-medication (for prevention of early adverse reaction)	snake antivenom + placebo/ no pre-medication	Early Adverse Reactions, other outcomes recorded and quality measures (as defined by trial authors)	September 2010
OTHER INTERVENTIONS TO MANAGE SNAKEBITE ENVENOMATION							
Toschlog 2013	To develop best practice guidelines for surgical interventions in the acute management of North American crotaline snake envenomation that are both evidence based and useful to the clinician	Not specified	North America	1. Early excision of tissue near bite site 2. methods for diagnosis of compartment syndrome 3. prophylactic fasciotomy 4. fasciotomy (curative for those with compartment syndrome)	1. standard care alone (including antivenom, if indicated) 2. NA 3. standard care alone (including antivenom, if indicated) 4. standard care alone (including antivenom, if indicated)	All late bleeding events reported in any study (specific definition by SR author).	July 2012

The SRs we found looked at the following aspects of management of snakebite envenomation:

- **First-aid for snakebite**: One SR looked comprehensively at all first-aid interventions for management of snakebites that is feasible for laypeople without medical background [21]. The SR had included 14 studies, of which two were randomised controlled trials (RCT), five were non-randomised intervention studies with control group, four were retrospective cohort studies, and three were prospective cohort design.
- **Effectiveness and safety of SAVs**: Six SRs evaluated different types of SAV for envenoming taking a snake species or genus specific approach [14, 16, 17, 19, 24, 25], while three took a more broad non-species-specific approach [20, 22, 23].

The SRs which took a snake-species specific approach included 81 studies of various designs. Among the studies which took a non-species-specific approach one was an empty review [23], while the other two SRs included 31 studies in total [20, 22].

- **Interventions to prevent adverse reactions due to SAV therapy:** Two SRs looked at interventions for preventing adverse drug reactions due to SAV therapy [13, 15]. Together, these two SRs included nine studies.
- **Other interventions for management of snakebite envenomation**: There was only one SR which evaluated surgical interventions for North American *Crotaline* snake envenomation [18]. It included 42 studies but did not report the total number of participants.

### Synthesis of findings from included systematic reviews on interventions

A narrative overview of the findings from the included SRs is presented in a structured manner based on typology of interventions.

#### First-aid for snakebite

The SR on first-aid [21] had a broad scope and included six different types of interventions. It included 1295 participants from 14 studies which were conducted in Australia (n = 4), Brazil (n = 2), India (n = 2), Myanmar (n = 2), Nigeria (n = 2), USA (n = 1) and China (n = 1).

- **Tourniquet**

The SR identified seven studies on effect of tourniquet on snakebite and found:

○ No significant differences between those treated with a tourniquet (with or without additional incisions in the bite wound) and victims who received no tourniquet or no first aid for death (Relative Risk (RR) 0.77; 95% CI 0.13 to 4.41); and the occurrence of death or disability (Odds Ratio (OR) 4.7; 95% CI 0.58 to 212).
○ No significant difference was seen between those treated with a tourniquet (irrespective of additional wound incisions),in comparison to those patients with snakebite who received no tourniquet or no first aid for the following outcomes: acute renal failure (RR 1.24; 95% CI 0.33 to 4.66) [22], acute respiratory failure (RR 1.4; 95% CI 0.3 to 6.53) [22], occurrence of haemorrhagic syndrome (RR 0.95; 95% CI 0.77 to 1.17) [30], and incidence of multiple organ dysfunction syndrome (RR 1.85; 95% CI 0.56 to 6.15).
○ Only a single study included in this SR had studied duration of hospitalisation and found no significant difference in the duration of hospital stay between snakebite victims treated with a tourniquet and those receiving no first aid (MD -0.3 days; 95% CI -1.9 to 1.3), another found a significant increase in the duration of hospital stay between snakebite victims treated with a tourniquet and those receiving no first aid (4.6±2.0 days vs 3.7±2.5 days; MD 0.9, p = 0.04).
○ Mixed evidence on wound related outcomes from different studies was found:
  ■ Increase in local swelling for those treated with a tourniquet (and no local incisions) (RR 1.71; 95% CI 1.49 to 1.96) and those treated with a tourniquet and wound incisions (RR 1.71; 95% CI 1.49 to 1.96), when compared to snakebite victims receiving no first aid.
  ■ Significantly increased odds for an increased severity of local envenomation in snakebite victims receiving a tourniquet, compared to those not receiving a tourniquet (OR 4.31; 95% CI 1.33 to 13.89).
  ■ No significant differences were found between snakebite victims treated with a tourniquet (with or without additional incisions in the bite wound) and victims who received no tourniquet or no first aid for tissue necrosis (RR 0.75; 95% CI 0.14 to 4.12) and local oedema (RR 0.98; 95% CI 0.6 to 1.61).

- **Incision of the bite wound**

The SR identified two studies on effect of incision of the bite wound and found:

○ No statistically significant difference in the incidence of death or disability (OR 4.3; 95% CI 0.18 to 275) between those whose bite wounds were incised as a part of first-aid and those receiving no first aid.
○ No difference in occurrence of haemorrhagic syndrome (RR 1.05; 95% CI 0.71 to 1.53), in comparison to those receiving no first aid.
○ Significantly increased incidence of local swelling upon incision (RR 1.66; 95% CI 1.40 to 1.97), in comparison to those receiving no first aid.
○ Significant decrease in the duration of hospitalisation in those whose snakebite wound was incised in comparison to those whose bite wound was not incised (2.9±1.6 days vs 4.6±2.2 days; MD -1.70 days; p = 0.03)

- **Suction of the bite wound**

The SR identified only one study which looked the effect of suction of bite-would and reported:

○ No significant increase in the occurrence of death or disability (RR 1.33; 95% CI 0.07 to 26.98) compared to patients who had not received first aid.
○ No significant increase in the duration of hospitalisation (median 6 days vs. 4 days, p = 0.7) compared to those who did not receive suction.

- **Snake stones**

The SR identified two studies on effect of snake stones (animal bones or stones used in folk and indigenous medicine for treatment of snakebite) and found:

○ No difference in the occurrence of death or disability between those treated with snake stones in comparison to those receiving no first aid (OR 13; 95% CI 0.39 to 823).
○ No significant decrease in duration of hospitalisation in those with snakebite patients who had applied snake stones in comparison to those not receiving any first-aid (MD -0.2; 95% CI -2.57 to 2.17) or in comparison to those not being treated by snake stones (median 2.5 days vs. 4 days; p = 0.09).

- **Traditional medicine and concoctions:**

The SR identified two studies that evaluated the use of traditional medicine and concoctions and found:

○ Statistically significant increased odds for death or disability in snakebite patients treated with concoctions applied to the bite wound, compared to those who had not applied concoctions to the wound (OR 15; 95% CI 1.4 to 708).
○ Statistically significant increase in odds for death or disability in snakebite patients who had ingested concoctions (6/10), compared to those who did not ingest (OR 20; 95% CI 1.4 to 963).
○ No significant decrease in the duration of hospitalisation in those who received traditional medicine, compared to those who did not received no first aid (MD 0.6 days; 95% CI -1.23 to 2.43). There was no difference in the duration of hospitalisation between those who were treated with concoctions applied to the bite wound, in comparison with those on whom no concoction was applied (median 5days vs. 4 days; p = 0.6), those who ingested concoctions, in comparison to those who did not ingest (median about 4 days in both; p = 0.84).

- **Pressure Immobilization**

The SR identified seven studies related to pressure immobilisation on snakebite but none of them reported any outcome of our interest.

#### Effectiveness and safety of SAVs: species or genus specific systematic reviews

Four SRs looked at evidence with respect to *Crotalidae* polyvalent immune Fab antivenom (FabAV) for *Crotalinae sp (*North American Pit Viper*)* envenomation. One looked specifically at children [24], one on those with severe envenomation [19], one on those who developed medically significant late bleeding [14] and another looked specifically at safety aspects [17]. This apart, two other SR looked at *Echis occelatus* envenomation in West Africa and *Vipera spp* envenomation in Europe [29, 30]. The evidence with these regards is summarised below:

○ ***Crotalidae* polyvalent immune fab (FabAV) in children**
  The SR found 10 studies (six case reports, three descriptive reports, and one RCT) with a total of 47 children [24]. When pooled the prevalence of adverse events was found to be in 8.5% of the children (4/47). Of these, three were acute reactions, and one was serum sickness on hospital discharge. All except two studies did not have any recurrent local effects (defined as progression of local injury after initial response to SAV) and late coagulopathy (defined as coagulopathy occurring after initial normal values). One study had 8% (1/12) recurrent local effects and 8% (1/12) late coagulopathy while another study had 75% (3/4) patients who had late coagulopathy.
○ **FabAV in those with severe envenomation**
  The SR found 19 studies consisting of 24 people with severe North American Pit Viper envenomation[19]. Seven cases were described in five cohort studies and 17 cases were described in 14 single patient case reports or non-cohort case series. Persistent severe venom effect (limb swelling, limb pain, soft tissue bleeding, thrombocytopenia, neurotoxicity, or compartment syndrome) was seen in 0% of patients in cohort study but 53% of patients in non-cohort reports. No patient developed systemic bleeding but recurrent and/or delayed-onset severe defibrination syndrome was found in patients.
○ **FabAV in those who develop medically significant late bleeding**
  The SR included 19 cohort studies (two cohorts were within the context of RCTs) consisting of 1017 patients. Late bleeding was seen in nine patients (0.9%; 95% CI 0.4% to 2.2%) with five patients developing medically significant late bleeding. (0.5%; 95% CI 0.1% to 1.7%) [14]. Eight of the nine patients who had late bleeding were cases of Rattlesnake envenomation. No deaths or sequalae of any kind was reported.
○ ***Safety of* FabAV for North American crotaline snake envenomation**
  The SR included 11 studies (seven retrospective studies, three prospective studies, and one that had both prospective and retrospective data) and included 661 participants [17]. The combined estimate of incidence of early hypersensitivity was 0.08 (95% CI 0.05 to 0.11). The pooled estimate of serum sickness incidence was 0.13 (95% CI 0.07 to 0.21) from amongst the seven studies which reported it.
○ **SAV for carpet viper (Echis ocellatus) envenoming in West Africa**
  The SR found 22 studies (four RCTs, 12 observational studies, and six preclinical studies) [16]. Pooled meta-analysis found that the odds of dying decreased by as much as 75% (OR 0.25; 95% CI 0.14 to 0.45) of dying among those treated with a specific antivenom compared to non-specific or no anti-venoms. Mortality rates were more than double when there was stock-out of reliable SAVs (RR 2.33; 95% CI 1.26 to 4.06).
○ **Anti-European Vipera spp antivenoms**
  The SR found 40 studies (excepting pre-clinical studies which were included) on various types of anti-European *Vipera spp* antivenoms involving about 2602 participants [25]. There were 14 studies each on Zagreb (n = 1306), and on ViperaTAb (n = 197), 11 studies on ViperFAV (n = 558), three studies on Biomed (n = 43), two studies on Bulbio antivenom (n = 69), and one case-report on Viekvin (n = 1). There were eight studies in the SR which did not specify the antivenom used.
  Deaths were reported only in patients given Zagreb SAV and the rate was 0.2% (n = 5). The median length of hospitalisation in patients who were given ViperFAV or ViperaTAb was significantly less than those being given IM Bulbio or Zagreb antivenoms (1 to 4.8 days versus 2 to 18 days).
  Adverse reactions were reported in 1.5% (37 of 2408 cases including 7 cases of anaphylaxis) 5%) in which SAV was administered. This varied between 0.5 to 2.0% in patients administered with ViperaTAb, Zagreb, and ViperFAV antivenom, 4.7% in those who received Biomed antivenom. No adverse reactions were reported in those administered Bulbio antivenom (n = 67) and in the single patient administered Viekvin antivenom.

#### Effectiveness and safety of SAVs: broad non -species/genus specific systematic reviews

There were three SRs which took a broad non-species / genus specific approach and investigated the role of SAVs in venom induced consumption coagulopathy in people with snakebite envenomation [23], effectiveness and safety of SAVs available commercially in sub-Saharan Africa [22], and, on different dosing regiments (low vs. high) of SAVs [20].

- **SAVs for managing venom induced consumption coagulopathy**

The SR on RCTs on this issue did not find any studies which met eligibility criteria [23].

- **SAVs available in sub-Saharan Africa**

The SR [22] took a phased approach, wherein the authors first conducted a market analysis to obtain a comprehensive list of SAVs available in the sub-Saharan Africa and then looked systematically for evidence (of any design) for these SAVs specific to the region. This is crucial because there is substantial intra-species variation based on climate and geography. The SR found 26 studies (two RCTs, five non-randomised comparative clinical studies, 11 observational cohort studies, and eight anecdotal clinical reports) on nine SAVs available in the sub-Saharan Africa.

The SR did not find any studies from sub-Saharan Africa on the following seven SAVs, although they were available in the markets:

○ ASNA antivenom–D (Bharat Serums and Vaccines)
○ Snake Venom Antiserum (PanAfrica) aka Premium-A (Premium Serums)
○ Snake Venom Antiserum (Central Africa) aka Premium-CA (Premium Serums)
○ Afriven 10, Snake Venom Antiserum (African) aka VINS-A (VINS Bioproducts)
○ Anti-Snake Venom Serum Central Africa aka VINS-CA (VINS Bioproducts)
○ Snake venom antiserum *Echis ocellatus* (VINS Bioproducts)
○ SAIMR-Boomslang (SAVP)
○ **EchiTabPlus (ICP) and EchiTabG (Micropharm)–**One RCT and two observational studies were found related to EchiTabPlus and EchiTabG for *Echis ocellatus* envenoming. For the RCT, exact difference in outcomes were not presented though the SR mentioned “ET-Plus was found to be a little more effective than an initial dose of one vial of EchiTabG, and a little less safe”[22]. Very low case-fatality was reported in the two observational studies from Nigeria and Central African Republic on use of EchiTabPlus or EchiTabG for *Echis ocellatus* envenoming. However, an early hypersensitivity reaction was seen in 21 patients (6.9%).
○ **Inoserp-Pan African (Inosan)-** The SR found two studies which found case fatality rates of 3.17% in Senegal and 4% and 0.92% in northern Benin and Guinea from a multicentre observational study with 8% of patients in whom adverse events were reported. The multi-country study from Benin and Guinea had many cases of *Echis ocellatus* in Benin. No specific species information was presented in the SR for the study from Senegal. Blood coagulability was found to be restored within 24 hours in 87.5% and 98% of patients in the respective studies.
○ **Fav-Afrique aka FAV-A(Sanofi Pasteur)**- FAV-A was studied in eight cohort studies from Cameroon (2/41 had minor adverse event; no death, no serum sickness), Ghana (mortality rate 1.8%), Chad (mortality rate 6.67%), Central African Republic (mortality rate 7.47% in a prospective study and 0.5% in a retrospective study), and Republic of Djibouti (no deaths or adverse events reported in three cohorts). The study in Cameroon and Central African Republic were conducted in an area where *Echis ocellatus was common*. *The three cohorts from Djibuouti found FAV-A to restore blood coagulability on Echis pyramidum* bites too. Only one patient in a single study from Djibouti which enrolled 31 patients had necrosis. No information about necrosis was reported in the SR for other studies.
○ **SAIMR-Polyvalent (SAVP)—**There were six studies on SAIMR-Poly in which a total of 5 deaths were seen in 144 included patients (death rate 3.47%). The SR noted varying rates of adverse events with one showing severe early (anaphylactoid) reaction in 76.47% patients. The adverse event rate across studies was between 10% to 15%.
○ **SAIMR Echis ocellatus / Echis Pyramidum (SAVP)—**There were three studies from Nigeria, of which one was an RCT. None of the three studies reported any deaths. The RCT in the SR found that SAIMR-Echis was more effective than SAIME Behringwerke in terms of reversing haematological abnormalities more rapidly (data not specifically reported). The RCT also noted early hypersensitivity in four out of 23 patients while one observational study found adverse reaction in 14 out of 48 patients (one study did not report adverse effects).
○ **Antivipmyn-Africa (Instituto Bioclon /Silanes)–**The SR found four studies which reported case fatality rates of 3.11% in Benin, 10% in Central Africa, 18.2% in Guinea, and 15.4% (low dose) and 17.6% (high dose) in another study in Guinea. A low rate of adverse events (between 10% to 15%) was reported across studies on Antivip-A.
○ **ASNA antivenom—C (Bharat Serums and Vaccines**)—There was one post-marketing surveillance study from Central Ghana which found 22% mortality and 7.58% anaphylactic shock. Another study included in the SR was from Nigeria and it reported that ASNA-C was ineffective in restoring blood coagulopathy and causing in allergic reactions in many cases. All the studies were conducted in areas where *Echis Occelatus* bites are common.
○ Vacsera **POLY-** One retrospective study from Ethiopia reported 17% deaths among 23 patients with prolonged clotting time who were treated with Vacsera Poly.

#### Different dosing regimens of SAVs

The SR [20] found five RCTs on low versus high dosage regiments of SAV, out of which four were from India and one from Brazil. However, the distinction used between low and high dosage was not specified a priori and as a consequent there were overlaps with low doses ranging from 20–220 ml while high dosage ranged from 40–550 ml. A volume-based classification of dosing regimens as done in this SR might also be inappropriate, because different antivenoms have different protein concentrations leading to differences in the amount of protein administered for the same volume[31].

Four trials reported mortality out of which one did not report any death. Pooled result from other three trails showed no significant difference in death between those with high and low doses of SAVs. (RR 0.69; 95% CI 0.38 to 1.26)

There was no significant difference in rates for neurological complications (RR 0.82; 95% CI 0.23 to 2.94), acute renal failure (RR 0.87; 95% CI 0.62 to 1.21), and bleeding or disseminate intravascular coagulation (RR 0.77; 95% CI 0.46 to 1.29). No significant difference was noted in time to normalisation of clotting time between high dose versus low dose group in one trial (10hours 23 minutes versus 9 hours) while another trial found a significant difference (20.67 ± 9.61 hours in high dose group (regimen I), 16.55 ± 9.84 hours in low dose (regimen II), and 13.4 ± 7.16 hours in low dose (regimen III)).

Adverse SAV reactions (itching, urticaria, and erythema) occurred in eight of 30 patients in the high dose group and 8 out of 60 patients in the low dose group in one trial. The other three trials did not report any major adverse events.

Duration of hospitalisation was reported from two studies and results were pooled to find that low-dose SAV led to 1.27 less days of hospitalisation compared to the high dose group (MD −1.27 days, −2.05 to − 0.5). Another study also reported duration of hospitalisation, but the SR could not pool the data due to non-reporting of standard deviation. It found "no difference in the average hospital stay (days) between the low dose and high dose (8.42 vs. 9.02 days).

The study calculated cost-effectiveness using prices of Indian polyvalent SAV prices. It stated that a low-dose regimen led to savings of INR 500–2000 (USD 10–140) excluding any other expenditures (including expenditure on hospitalisation, and other therapies).

#### Interventions to prevent adverse reactions due to SAV therapy

Two SR investigated interventions to prevent adverse reactions to SAV administration [13, 15]. The study published earlier [13] included only two RCTs from Brazil and Sri Lanka while Habib 2011 [15] included three RCTs and four cohort studies. The SRs found:

- **Prophylactic medication to prevent early adverse reaction (EAR)**
  The seven studies that Habib et al [15] included had 10 comparisons of adrenaline alone or in combination, hydrocortisone alone, anti-histamine alone or in combination with steroids. The overall pooled RR for any prophylactic pre-medication to no pre-medication for EAR was 0.70 (0.50 to 0.99) but there was high heterogeneity implying different effects of particular types of pre-medications.
- **Prophylactic Adrenaline**
  Nuchpayoon et al [13] included only one trial from Sri Lanka which found that those who received adrenaline had significantly lesser adverse allergic reactions to SAV (Haffkine poly-specific) overall (RR 0.25; 95% CI 0.11 to 0.57) than those receiving placebo. The trial had also noted that severe reactions were many times more in the placebo group over the adrenaline group (RR 0.10; 95% CI 0.01 to 1.77). No death was recorded in either of the groups. No patient developed hypertension (blood pressure >160/100 mmHg), arrhythmia (other than sinus tachycardia), or neurological deficits suggestive of cerebrovascular accidents in either of the groups.
  Habib 2011 [15] had included three studies (including the Sri-Lankan trial which was included in Nuchprayoon) on adrenaline-containing pre-medication (adrenaline alone or with promethazine/hydrocortisone) and found a risk-ratio of 0.32 (95% CI 0.18 to 0.58) with no heterogeneity, when compared to no pre-medication or placebo. The other two studies were a retrospective cohort from Papua New Guinea and nested cohort from Australia with risk-ratio of 0.27 (0.10, 0.79) and 0.78 (0.21, 2.90) respectively for subcutaneous adrenaline-containing pre-medication compared to no pre-medication.
- **Prophylactic Steroid**
  While Nuchprayoon did not find any studies which had looked at the role of steroid alone, Habib found one RCT from Sri Lanka which found no difference for development of EAR between use of hydrocortisone and placebo (RR 0.98; 95% CI 0.70 to 1.39) and the trial was prematurely stopped [15].
- **Prophylactic Anti-Histamine**
  Both SRs found one trial from Brazil on Bothrops envenomation patients to prevent reactions due to *Bothrop* specific SAV (three manufactures: Instituto Butantan, Fundaçao Ezequiel Dias, or Instituto Vital Brazil) and found no difference in acute reactions between those who received promethazine and those who did not (RR 0.98; 95% CI 0.50 to 1.93). One patient from each treatment group suffered severe anaphylaxis. No death was reported in either of the groups.
- **Prophylactic Steroid along with anti-histamine**
  Habib [15]found five studies which had explored several combinations of prophylactic steroid with different anti-histamine and although separate pooled RR for this was not reported, it mentioned that the result was not statistically significant and there were issues with heterogeneity, paucity and quality of data.

#### Other interventions for management of snakebite envenomation

There was only one SR under this category which was done in the context of consensus-based recommendations being developed for surgical consideration for North American Pit Viper (*Crotalinae*) envenomation [18]. It found evidence on several key issues, one of which pertained to diagnostic accuracy issues and hence not of interest (diagnostic criteria for compartment syndrome) to this overview:

- **Early excision of tissue near bite in *Crotaline spp*. envenomation**
  The SR found two old observational studies (with no comparison group) when early excision along with tourniquet and ice-water immersion but not SAV being administered typically showed worse tissue outcomes (not exactly specified). In the modern context, where SAV administration is the norm, the review found no comparative clinical trials which had examined role of early excision (alone or as an adjunct with SAV). It however, found 16 studies which showed excellent results (outcomes were not explicitly stated) with SAV without incisions or excisions in comparison to just one study which found to the contrary. The SR found no literature in relation to debridement of necrotic tissue or in relation to management of puncture wounds on tendon sheaths for patients with snake envenomation.
- **Prophylactic fasciotomy for preventing compartmental syndrome in Crotaline spp envenomation**
  Prophylactic fasciotomy (done before compartment syndrome develops in *Crotaline spp*) alone or in combination with standard therapy including SAV was found to not improve outcomes. The outcomes were not explicitly specified but are related to "scarring and wound-healing" and "elevated compartment pressure". The quality of evidence was determined to be moderate by the consensus group and was based on two human and one porcine study.
- **Therapeutic Fasciotomy for treating compartmental syndrome in Crotaline spp envenomation**
  It was found that FAb SAV administration decreased myonecrosis and decreased the need for fasciotomy. Therapeutic fasciotomy in those with diagnosed compartmental syndrome for *Crotaline spp* envenomation was found to not decrease intra-compartmental pressure as per a recent evidence-based review included in the SR. However, despite this, the consensus committee mentioned about a “large body of evidence supporting fasciotomy in compartment syndrome caused by fractures, crush injuries, and electrical burns, it is logical that fasciotomy should be performed in cases where aggressive antivenom therapy fails to correct impaired tissue perfusion.” The evidence was not cited, while a recommendation was made for therapeutic fasciotomy through an algorithm developed by the consensus panel.

### Quality of primary studies included in systematic reviews

Seven included SRs did not conduct any quality appraisal of included studies [14, 18, 19, 22, 24, 25, 30]. The study on low-dose versus high dose of SAV reported that they used the Cochrane tool and reported that the included trials were of “moderate quality” [20]. The study to understand safety of FabAV [17] used the Jadad scale for RCTs, Newcastle-Ottawa Quality Assessment Scale for observational prospective studies, and a chart review tool for retrospective studies. The Jadad score for the included RCT had an Endorsement Frequency of 84.5%, all the prospective cohort studies had a score of 7 out of 9 (9 being lowest risk of bias) while the retrospective studies had varying quality.

Quality of evidence on different outcomes were reported to be measured by GRADE approach in only two SRs [20, 21] and in both the SRs. the quality of outcomes was found to be low or very low.

### Confidence in results of included SRs

**Fig 2:**
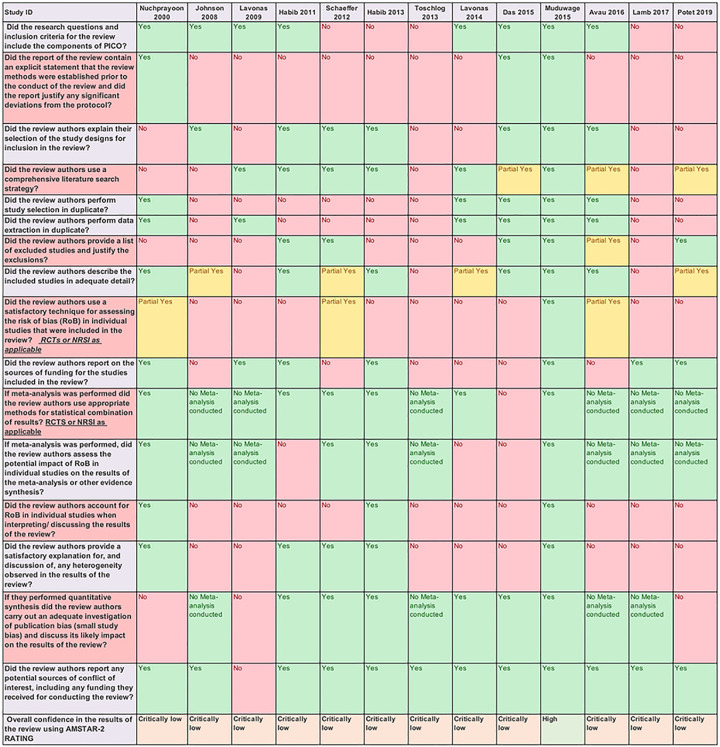
AMSTAR-2 ratings showing confidence in results of included systematic reviews.

We used AMSTAR-2 for assessing the confidence in results of included SRs and found that except for one [23], all were rated to have critically low confidence in results. This implies the SR had more than one critical flaw and should not be relied on to provide an accurate and comprehensive summary of the available primary studies on the topic. We rated Maduwage et al. [23] to have high overall confidence in the results of the SR. AMSTAR-2 ratings for the included SRs are summarised in Fig 2.

## Discussion

### Summary of main results

The available evidence from 13 completed SRs related to management of snakebite envenomation covers a range of interventions (pharmacological and otherwise) and in diverse settings (geographical, species specific, and otherwise). While effect estimates vary, it is evident that there are glaring gaps in terms of availability and quality of evidence. We have summarised the summary evidence for all the interventions at synthesis level in Table 2. Largely we see that high-quality review-level evidence is not available for almost all intervention-outcome pairs. There is no synthesised evidence with regards to quality of life or mental health outcomes across the board and only few SRs [20, 25, 32] had envisaged to understand the effect of interventions to decrease health systems burden (through cost or duration of hospitalisation).

**Table 2:** Summary of evidence for interventions for management of snakebite from systematic reviews (SR) (Colour code key at bottom).

BROAD DOMAIN	Intervention Versus Comparator (if available)	No. Of Studies	Summary direction of evidence for Primary Outcome	Summary direction of evidence for secondary outcome
**FIRST AID**	Tourniquet versus No tourniquet/first aid	7	• Death–no difference • Acute renal failure–no difference • Acute respiratory failure–no difference • Occurrence of hemorrhagic syndrome–no difference • Incidence of multiple organ dysfunction syndrome–no difference	• Duration of hospital stay–heterogeneity in results • Wound related outcomes • Increase in local swelling–**tourniquet not effective** • increased severity of local envenomation–**tourniquet not effective** • Necrosis–no difference • Local Oedema–no difference • Occurrence of death or disability (composite)- no difference
	Incision of the bite wound versus No first aid/incision	2	Occurrence of haemorrhagic syndrome–no difference	• Duration of hospitalisation–**Incision effective** • Increased incidence of local swelling–**Incision not effective** • Incidence of death or disability (composite)–no difference
	Suction of the bite wound versus No first aid/suction	1	No outcome of interest reported	• Duration of hospitalisation–**suction not effective** • Occurrence of death or disability (composite)–**suction not effective**
	Snake stones versus No first aid/stone stones	2	No outcome of interest reported	• Duration of hospitalisation–**snake stones not effective** • Occurrence of death or disability (composite)–no difference
	Traditional medicines and concoctions versus No first aid/concoctions	2	No outcome of interest reported	• Duration of hospitalisation–**traditional medicine and concoctions not effective** • Occurrence of death or disability (composite)–**concoctions not effective**
	Pressure immobilisation	7	No outcome of interest reported	No outcome of interest reported
**EFFECTIVENESS AND SAFETY OF SAVS (species or genus specific SRs)**	Crotalidae polyvalent immune Fab (FabAV) (in children)	10	• Adverse events (acute reactions, serum sickness)–**FabAV effective** • Late coagulopathy–**FabAV effective**	Recurrent local effects (local injury)–**FabAV effective**
	FabAV (in those with severe envenomation)	19	• Persistent severe venom effect (limb swelling, limb pain, soft tissue bleeding, thrombocytopenia, neurotoxicity, or compartment syndrome)–heterogeneity in study results • Systemic bleeding–**FabAV effective** • Recurrent and/or delayed-onset severe defibrination syndrome–**FabAV not effective**	No outcome of interest reported
	FabAV (in those who develop medically significant late bleeding)	19	• Late bleeding–**FabAV lead to low rates of medically significant late bleeding an** • Specific death—**No deaths or permanent sequale due to bleeding in FAbAV treated**	No outcome of interest reported
	Safety of FabAV (in patients of North American crotaline envenomation)	11	• Early hypersensitivity–**FabAV safe** • Serum sickness—**FabAV safe** • Deaths as a result of serum sickness specifically reported- **FabAV safe**	No outcome of interest reported
	Specific SAV (for carpet viper envenoming in West Africa) Versus non-specific or no anti-venoms	22	• Mortality–**Specific SAV effective**	No outcome of interest reported
	Comparison between different types of Anti-European Vipera spp antivenoms	40	• Death—**Zagreb antivenom not effective** in reducing deaths compared to other anti-European *Vipera spp*• Adverse reactions—**ViperaTAb, Zagreb, and ViperFAV had less** adverse reactions compared to Biomed, Bulbio and Viekvin antivenom.	Duration of hospitalisation—**ViperFAV or ViperaTAb antivenoms more effective** compared to Bulbio or Zagreb antivenoms.
**EFFECTIVENESS AND SAFETY OF SAVS** **(broad non -species/genus specific SRs)**	SAVs (for managing venom induced consumption coagulopathy)	0	No Evidence Found	No Evidence found
	Comparisons between various types of SAVs available in sub-Saharan Africa	26	• Mortality–**EchiTabPlus or EchiTabG, Inoserp-Pan African (Inosan), SAIMR Echis ocellatus effective** in reducing mortality. Heterogeneity in results on Fav-Afrique aka FAV-A administration. Antivipmyn-Africa antivenom, ASNA antivenom and Vascera POLY ineffective in reducing mortality. • Blood coagulopathy—**ET-Plus effective** in restoring blood coagulopathy compared to ET- G. **Inoserp-Pan African (Inosan) effective while ASNA antivenom–C ineffective** in restoring blood coagulopathy • Adverse events—**ET-Plus a little less safe** than an initial dose of one vial of EchiTabG. **Inoserp-Pan African (Inosan) effective** in reducing adverse events. Rate of adverse events high in SAIMR Polyvalent. Lower rate of adverse reactions was reported by Antivipmyn-Africa antivenom in comparison to SAIMR Echis. The rate of severe adverse events appeared to be high in ASNA antivenom–C. • Haematological abnormalities–**SAIMR-Echis more effective than SAIME Behringwerke antivenom** for reversing haematological abnormalities • Neurotoxicity—Antivipmyn-Africa antivenom showed poor results	No outcome of interest reported
	High dose of SAV versus Low dose of SAV	20	• Mortality–no difference • Neurological complications–no difference • Acute renal failure–no difference • Bleeding or disseminate intravascular coagulation–no difference • Adverse reactions (itching, urticaria, and erythema)–**low dose effective**	• Duration of hospitalisation–heterogeneity of results • Cost-effectiveness–**Low dose more cost effective**
**INTERVENTIONS TO PREVENT ADVERSE REACTIONS DUE TO SAV THERAPY**	Prophylactic pre-medication Versus No pre-medication	10	Early adverse reactions–**pre-medication effective** (high heterogeneity in implying effects of different pre-medications)	No outcome of interest reported
	Prophylactic Adrenaline versus Placebo/no premedication	4	Early adverse reactions–**Adrenaline effective in prevention**	No outcome of interest reported
	Prophylactic hydrocortisone versus Placebo	1	• Early adverse reactions–no difference	No outcome of interest reported
	Prophylactic promethazine versus No premedication	1	• Early adverse reactions including anaphylaxis–no difference	No outcome of interest reported
	Prophylactic Steroid along with Anti-histamine versus Only Anti-histamine (different types)	5	• Early adverse reactions–no difference	No outcome of interest reported
**OTHER INTERVENTIONS**	Early excision of tissue near bite (in Crotaline spp. envenomation)	19	No outcome of interest reported	• Worse tissue outcomes—Early excision along with tourniquet and ice-water immersion but not with SAV being administered **not effective**
	Prophylactic fasciotomy (in Crotaline spp envenomation) Versus standard care alone (including antivenom)	3	No outcome of interest reported	• Outcomes related to "scarring and wound-healing" and "elevated compartment pressure"—**Prophylactic fasciotomy not effective**
	Therapeutic Fasciotomy (in Crotaline spp envenomation)	NR	• No outcome of interest reported	No outcome of interest reported

Colour coding based on AMSTAR-2 appraisal–Peach: Critically Low confidence in evidence from SR. Green: High confidence in evidence from SR

We found that evidence for several key aspects regarding first aid for snakebite envenomation is required. Evidence pertains to only a few studies and with small number of participants [32]. Low quality evidence exists that tourniquet, incision, suction, snake stones and traditional medicines and concoctions are not effective for several outcomes, although evidence on several key outcomes is not available or show no difference compared to their non-application for first aid. There is no evidence on pressure immobilisation related to outcomes of interest.

Evidence with respect to specific geographic settings and for many specific anti-venoms is unavailable at the synthesis level and also at the primary study level (as for example in Africa [22]). Despite SAV being the only life-saving intervention for snakebite dosing regimens and their safety and effectiveness, key clinical issues are studied only in a handful of trials–the evidence base thus being low quality, inconclusive and not providing contextual information [20, 33]. Evidence related to late adverse reactions, wound-related outcomes, quality of life, duration of hospitalisation, costs and disability is scarcely available. Prophylactic medications for preventing adverse reactions for SAV has been studied in only a few RCTs and there is some evidence on the effectiveness and safety of adrenaline for this purpose [13, 15]. There is no evidence suggesting the use of steroids, anti-histamines or their combination for preventing adverse reactions. The SRs on species-specific treatment issues (including SAVs and role of surgical interventions) are mostly restricted to North American Pit Viper (*Crotalidae*) and Carpet Viper (*Echis occelatus*) envenomation [14, 18, 24, 34, 35]. The FabAV antivenom is found to be effective in many studies for children, for those with severe envenomation and for those who develop medically significant late bleeding). It has been found to be safe in several studies. Specific SAV for Carpet Viper envenoming in West Africa is more effective in decreasing mortality compared to non-specific SAVs or no SAVs. There is no synthesised evidence pertaining to envenomation due to other snake species specifically.

### Overall completeness and applicability of evidence

All except one SR were rated to have critically low-quality using AMSTAR-2 –this is a major cause of concern for evidence synthesis for snakebite. The only high quality review was an empty review [23], implying high confidence that there is no evidence for effectiveness and safety of SAV for neuromuscular paralysis. Key critical issues in the included SRs were lack of prior registration and/or publication of protocol, non-provision of list of excluded studies at full-text level, and non-usage of appropriate risk of bias tools and/or its usage to interpret results and discussion.

Most SRs did not assess the quality of included primary studies. Critical appraisal of included primary studies is a standard component of systematic reviews as it helps assess the quality of evidence. It enables decision makers to understand the level of confidence one might have in the results of the primary study. Even reviews which used risk of bias tools for critical appraisal of tools did not appropriately report the use of the tools, and the use of risk of bias/GRADE for drawing conclusions were not appropriate. Potet et al. [22] had planned to use the Newcastle Ottawa Scale to assess quality but abandoned their plan citing that the tool was “not well adapted to the overall very low quality of selected studies” and instead used a study-design based criterion. The Newcastle Ottawa Scale is, in fact, designed to assess quality of non-randomised studies. Several design aspects, beyond study design such as validity of measurements and blinding of outcome assessments, the quality of the conduct of the study (e.g. loss to follow up and success of blinding), absolute and relative size of any effects seen etc. are known to affect the quality of evidence. [36] This means that conclusions drawn from the SRs in terms of some products have “been tested in robust clinical studies and found effective”[22] needs to be cautiously interpreted. Application of risk of bias tool was also inappropriate in Das et al. [20] This review reported quality or risk of bias as “moderate degree as most were open label trials” by using the Cochrane Risk of Bias tool–without providing any further information. The Cochrane tool assessed a trial with ratings for low risk, high risk or uncertain risk for each of the six separate domains without a composite degree of bias being evaluated for individual RCTs. [37] Where quality of reported studies was mentioned, certainty of the effect estimates for different interventions included in the SRs varied but were almost never of high quality. Accounting for the impact of risk of bias of included primary studies in the results of the synthesis and accounting for it while interpreting the results of the SR would enable more informed decision in the future.

The current study also highlights two important aspects with respect to the completeness of the available evidence at the systematic review–there are many important interventions and outcomes on snakebite management on which SRs have not been conducted, and, for when they have been done, apart from quality of SR, there is need to update them. A full discussion on these aspects comprehensively is beyond the scope of the current study and the need for future work to guide this has been discussed subsequently. Broadly, some domains on which primary research evidence exists but no SR available or there is need for update available ones are—wound management, managing psychological impacts, role of antibiotics, interventions for preventing adverse reaction due to SAV and effectiveness of SAVs. [38–43]

### Potential biases in the overview process

The overview includes SRs irrespective of study design, recognising the fact that randomised evidence for snakebite envenoming might be difficult to generate. We used a comprehensive search strategy that was implemented in multiple electronic databases. Screening, data extraction and quality assessment using AMSTAR-2 was done by at least two study authors independently with discrepancy being resolved by consensus. As such, high rigor has been maintained in the overview process. The only limitation of our overview is that its broad scope has meant that we had to depend on the findings of SRs on varied topics without any consistent methods of reporting.

### Implications for practice, policy and research

With the development of WHO strategy and the goal to reduce death and disability due to snakebite envenomation to half by 2030, accentuated attention. [8] In our previous work, we evaluated WHO guidelines on snakebite envenomation and found limited use of available evidence in formulating recommendations and heavy reliance on expert opinion. [9] The current work highlights the challenges in formulating high quality evidence informed guidelines owing to the lack of high quality SRs. As such, the lack of high-quality SRs on snakebite is a critical gap which needs attention from global health funders. High-quality SRs and other evidence synthesis which can aid clinical and public health decision making and appropriate investments can guide future primary research too. Given the paucity in primary research evidence, conduct of RCTs and its resourcing is also needed. Developing an evidence gap map of RCTs for snakebite envenomation might be the first step towards this purpose to enable set research priorities. Our overview also indicated the lack of consistency in defining and measuring outcomes for snakebite envenoming. Standardisation on what outcomes are measured and how they are measured will enable comparison between different interventions and ensure relevance for different stakeholders including patients. There is a tremendous need for development of a core outcome set [44] for clinical studies on snakebite. The variation in species distribution as well as intra-species variation in venom composition implies the need for conduct of region, nation or state (sub-national) specific RCTs and SRs on different SAVs and their dosing regimens. The results of this overview can inform priorities for funding and conduct of high-quality SRs and other evidence synthesis on management of snakebite envenomation. Key considerations for practice, policy and research and policy is summarised in **Box 1**.

**Box 1: Key considerations for practice, policy and research**

- High quality systematic reviews to inform clinical practice guidelines do not exist. There is no strong evidence to either support or refute many interventions related to snakebite envenomation.
- Investments in "research on research” and evidence synthesis including conduct of high-quality systematic review, development of intervention evidence gap map, and development of core outcome sets on snakebite envenomation might help inform research policy and practice better.
- Systematic reviews on snakebite envenomation should follow high quality standards to enable critical assessment of existing evidence base for development of clinical practice guidelines.
- Systematic reviews on snakebite should extract snake-species specific data whenever reported. Even if species disaggregated outcome data is not reported in the primary studies, sub-group analysis might provide potentially useful information.
- Randomised controlled trials, providing evidence on effectiveness and safety of different snake anti-venoms specific in different geographic settings and for specific snake-species is a gap that needs to be addressed. Such trials should minimally use core-outcome sets to enable wider utility.
- Funding high quality randomised controlled trials addressing existing clinical issues on first-aid, different snake anti-venoms, preventing adverse drug reactions, and wound management for snakebite envenomation is a priority area that needs to be addressed.

## Conclusion

Ensuring safe, effective treatments which can bring down the burden of snakebite requires conduct of high-quality SRs. The lack of high-quality SRs hampers guideline development as well as informing priorities for primary research on snakebite.

## Supporting information

S1 Table PRISMA Checklist for Interventions for the management of snakebite envenoming: an overview of systematic reviews. (DOC)

S2 Table Reasons for exclusion in full-text phase for Interventions for the management of snakebite envenoming: an overview of systematic reviews. (DOCX)

S1 Text Search Strategies for Interventions for the management of snakebite envenoming: an overview of systematic reviews. (DOCX)
