# Supplementary material for: Interventions for the management of snakebite envenoming: An overview of systematic reviews
Source: PLoS Negl Trop Dis. 2020 Oct 13;14(10):e0008727. doi: 10.1371/journal.pntd.0008727 (PMC7584233; doi:10.1371/journal.pntd.0008727)
Supplement: S2 Table — (DOCX) [file pntd.0008727.s002.docx]

# S3 Table: Reasons for exclusion in full-text phase for Interventions for the management of snakebite envenoming: an overview of systematic reviews

| **Study Reference** | **Reason for exclusion in the overview** |
| --- | --- |
| 1. Silva LMG, Zamuner LF, David AC, et al. Photobiomodulation therapy on Bothrops snake venom-induced local pathological effects: a systematic review. Toxicon 2018;152:23-29. doi: http://dx.doi.org/10.1016/j.toxicon.2018.07.006 | Did not include studies conducted in human participants. |
| 1. Thomas Lamb, Michael Eddleston, Nwe MT. A review of the 20-minute whole blood clotting test at detecting coagulopathy from snake bite 2020 [Available from: <https://www.crd.york.ac.uk/prospero/display_record.php?ID=CRD42020168953>. | Not an intervention systematic review |
| 1. Mullins ME, Gerardo CJ, Lavonas EJ. Incidence of hypersensitivity reactions following copperhead snakebites treated with Fab AV or placebo. Toxicology letters 2016;259:S140. | Is a randomised controlled trial |
| 1. Brown SA, Seifert SA, Rayburn WF. Management of envenomations during pregnancy. Clinical Toxicology 2013;51(1):3-15. doi: http://dx.doi.org/10.3109/15563650.2012.760127 | Not an intervention systematic review |
| 1. Sarmiento K, Rodríguez A, Quevedo-Buitrago W, et al. Comparación de la eficacia, la seguridad y la farmacocinética de los antivenenos antiofídicos: revisión de literatura. Univ Med 2020;61(1):30-51. doi: 10.11144/javeriana.umed61-1.anti | Wrong outcome ( only pharmacokinetic outcomes reported) |
| 1. Mise YF, Lira-da-Silva RM, Carvalho FM. Time to treatment and severity of snake envenoming in Brazil. Rev Panam Salud Publica 2018;42 doi: 10.26633/rpsp.2018.52 | Not a systematic review |
| 1. González Manrique G, Motta O, Ramírez C, et al. Oftalmoplejía asociada a neurotoxicidad por veneno de serpiente: presentación de un caso y revisión de la literatura. Acta Neurol Colomb 2016;32(4):314-19. doi: 10.22379/24224022114 | Not a systematic review |
| 1. Piñeiro Pérez R, Carabaño Aguado I. Manejo práctico de mordeduras en Atención Primaria y en nuestro medio. Rev Pediatr Aten Primaria 2015;17(67):263-70. | Not a systematic review |
