# Supplementary material for: Interventions for the management of snakebite envenoming: An overview of systematic reviews
Source: PLoS Negl Trop Dis. 2020 Oct 13;14(10):e0008727. doi: 10.1371/journal.pntd.0008727 (PMC7584233; doi:10.1371/journal.pntd.0008727)
Supplement: S1 Text — (DOCX) [file pntd.0008727.s003.docx]

# S2 Table : Search Strategies for Interventions for the management of snakebite envenoming: an overview of systematic reviews

**Ovid MEDLINE(R) and Epub Ahead of Print, In-Process & Other Non-Indexed Citations, Daily and Versions(R)**

Search Strategy: <1946 to May 14, 2020>

--------------------------------------------------------------------------------

1 exp Snake Bites/ (4592)

2 Snakebit*.mp. (2221)

3 ((bite* or envenom*) adj3 Snake*).mp. (5710)

4 1 or 2 or 3 (6210)

5 exp Meta-Analysis/ (114638)

6 exp "Systematic Review"/ (127489)

7 (systematic review or meta-analysis).ti. (178380)

8 (systematic review or meta-analysis).ab. (185345)

9 ("systematic review" or "integrative review" or "rapid review" or "umbrella review").mp. (179799)

10 5 or 6 or 7 or 8 or 9 (273949)

11 4 and 10 (33)

***************************

**Global Health (EBSCOHost)**

Search Strategy: <1910 to 2020 Week 18>

--------------------------------------------------------------------------------

1 exp Snake Bites/ (3005)

2 Snakebit*.mp. (1644)

3 ((bite* or envenom*) adj3 Snake*).mp. (3751)

4 1 or 2 or 3 (4011)

5 exp Meta-Analysis/ (26938)

6 exp "Systematic Review"/ (29723)

7 (systematic review or meta-analysis).ti. (35139)

8 (systematic review or meta-analysis).ab. (36688)

9 ("systematic review" or "integrative review" or "rapid review" or "umbrella review").mp. (29700)

10 5 or 6 or 7 or 8 or 9 (51459)

11 4 and 10 (18)

***************************

**Embase Classic + Embase**

Search Strategy: <1947 to 2020 May 14>

--------------------------------------------------------------------------------

1 exp Snake Bites/ (6664)

2 Snakebit*.mp. (7325)

3 ((bite* or envenom*) adj3 Snake*).mp. (4177)

4 1 or 2 or 3 (8204)

5 exp Meta-Analysis/ (187025)

6 exp "Systematic Review"/ (244404)

7 (systematic review or meta-analysis).ti. (218593)

8 (systematic review or meta-analysis).ab. (239294)

9 ("systematic review" or "integrative review" or "rapid review" or "umbrella review").mp. (315624)

10 5 or 6 or 7 or 8 or 9 (432150)

11 4 and 10 (67)

12 limit 11 to exclude medline journals (8)

***************************

**EBM Reviews - Cochrane Database of Systematic Reviews, Database of Abstracts of Reviews of Effects, Cochrane Clinical Answers, Cochrane Central Register of Controlled Trials, Cochrane Methodology Register, Health Technology Assessment , NHS Economic Evaluation Database**

Search Strategy: Cochrane Database of Systematic Reviews <2005 to May 14, 2020>, EBM Reviews - ACP Journal Club <1991 to April 2020>, EBM Reviews - Database of Abstracts of Reviews of Effects <1st Quarter 2016>, EBM Reviews - Cochrane Clinical Answers <April 2020>, EBM Reviews - Cochrane Central Register of Controlled Trials <April 2020>, EBM Reviews - Cochrane Methodology Register <3rd Quarter 2012>, EBM Reviews - Health Technology Assessment <4th Quarter 2016>, EBM Reviews - NHS Economic Evaluation Database <1st Quarter 2016>

--------------------------------------------------------------------------------

1 exp Snake Bites/ (84)

2 Snakebit*.mp. (98)

3 ((bite* or envenom*) adj3 Snake*).mp. (149)

4 1 or 2 or 3 (173)

5 exp Meta-Analysis/ (23)

6 exp "Systematic Review"/ (0)

7 (systematic review or meta-analysis).ti. (40521)

8 (systematic review or meta-analysis).ab. (18987)

9 ("systematic review" or "integrative review" or "rapid review" or "umbrella review").mp. (58934)

10 5 or 6 or 7 or 8 or 9 (69887)

11 4 and 10 (8)

***************************

**APA PsycInfo**

Search Strategy: <1806 to May Week 2 2020>

--------------------------------------------------------------------------------

1 exp Snake Bites/ (0)

2 Snakebit*.mp. (17)

3 ((bite* or envenom*) adj3 Snake*).mp. (29)

4 1 or 2 or 3 (42)

5 exp Meta-Analysis/ (4673)

6 exp "Systematic Review"/ (321)

7 (systematic review or meta-analysis).ti. (31897)

8 (systematic review or meta-analysis).ab. (38182)

9 ("systematic review" or "integrative review" or "rapid review" or "umbrella review").mp. (29271)

10 5 or 6 or 7 or 8 or 9 (51280)

11 4 and 10 (0)

***************************

**CINAHL by EBSCO-Host**

Search Strategy: Saturday, May 16, 2020 9:39:32 AM

1. ( (MM "Snake Bites") OR Snakebit* OR ((bite* or envenom*) adj3 Snake*) ) AND ( (MM "Systematic Review") OR (MM "Meta Analysis") OR TI (systematic review OR meta-analysis) OR TI (systematic review OR meta-analysis) OR TX ("systematic review" or "integrative review" or "rapid review" or "umbrella review") )(17)

2. Limiters - Exclude MEDLINE records (9)

**Campbell Library**

Search Strategy: Saturday, May 16, 2020 9:39:32 AM

1. snake OR snakebite OR snake-bite OR envenomation (0) [in keywords Advance Search]

***************************

**SCIELO**

Search Strategy : 04 August 2020

1. (mordida de cobra) OR (mordedura de serpiente) Filter: todos los indices (All Indexes) - - 38 hits
